# Antiphospholipid Syndrome in Orthopaedic Foot and Ankle Surgery: A Propensity-Matched Analysis

**DOI:** 10.1177/24730114251393650

**Published:** 2025-11-29

**Authors:** Kush Mody, Avani A. Chopra, Michael Greenberg, Darian Napoleon, Tyler Stewart, Michael Aynardi, Sheldon Lin

**Affiliations:** 1 Rutgers New Jersey Medical School, Newark, NJ, USA; 2 Penn State College of Medicine, Hershey, PA, USA; 3 Atlantic Health System, Summit, NJ, USA

**Keywords:** antiphospholipid syndrome, foot and ankle surgery, thromboembolic risk, hypercoagulable state

## Abstract

**Background:**

Antiphospholipid syndrome (APS) is a systemic autoimmune disorder associated with a heightened risk of thromboembolic events. The purpose of this study is to evaluate the impact of APS on medical and surgical postoperative outcomes following foot and ankle surgery.

**Methods:**

Using the TriNetX Research Network, we identified patients undergoing foot and ankle surgery between 2004 and 2024. APS patients with and without postoperative venous thromboembolism (VTE) prophylaxis were matched 1:1 with controls based on demographics and comorbidities. Outcomes within 30 and 90 days postoperatively were compared, including thromboembolic events. Preoperative laboratory test values were also assessed in a subset of APS patients not on anticoagulation.

**Results:**

At 90 days postoperatively, APS patients receiving VTE prophylaxis (APS+VTE) (n = 524) had significantly higher rates of deep vein thrombosis (13.4% vs 9.0%, *P* = .024), but similar rates of stroke and pulmonary embolism compared to patients without APS receiving VTE prophylaxis (Control+VTE). Anemia was significantly less common in the APS+VTE group at 30 days (14.7% vs 22.3%, *P* = .001) and 90 days (16.4% vs 23.9%, *P* = .003). APS patients without postoperative anticoagulation (APS–VTE) (n = 932) had a higher rate of pulmonary embolism at 90 days (5.8% vs 3.3%, *P* = .011) compared to patients without APS and VTE prophylaxis (Control–VTE). Anticoagulation-naïve APS patients demonstrated significantly higher preoperative International Normalized Ratio (1.64 vs 1.09, *P* < .001), prothrombin time (17.7 vs 12.3, *P* < .001), activated partial thromboplastin time (38.6 vs 29.2, *P* < .001), serum creatinine (1.08 vs 0.94, *P* < .001), estimated dry weight (14.3 vs 13.7, *P* < .001), along with urea nitrogen (17.0 vs 15.9, *P* < .001), sodium (139.13 vs 139.25, *P* = .001), potassium (4.14 vs 4.10, *P* = .012), and bicarbonate (26.2 vs 25.8, *P* < .001) compared with matched controls.

**Conclusion:**

APS patients undergoing foot and ankle surgery had significantly higher thromboembolic event rates than controls, even with anticoagulation, and those who received anticoagulation had lower anemia rates, suggesting differential bleeding risk.

**Level of Evidence:**

Level III, retrospective cohort study.

## Introduction

Antiphospholipid syndrome (APS) is a systemic autoimmune disorder that may present as a primary condition or in association with other autoimmune diseases, most commonly systemic lupus erythematosus (SLE).^7,13^ The diagnosis of APS requires the presence of a clinical criterion (thrombosis and/or pregnancy morbidity) and laboratory criterion of antiphospholipid antibodies (aPL).^12,15^ Antiphospholipid antibodies include lupus anticoagulant, anticardiolipin, and anti-β2-glycoprotein I, which activate prothrombotic pathways and predispose patients to arterial, venous, and small vessel thrombosis.^7,13^ Surgical intervention is known to induce a hypercoagulable state, and when combined with the underlying thrombogenic profile of APS, may further elevate the risk of postoperative complications in this population.^2,4^

Although evidence remains limited, existing literature suggests an increased risk of thromboembolic events in patients with APS undergoing surgery.^1,9,17^ A systematic review of patients with APS and SLE undergoing cardiac surgery reported higher rates of postoperative thrombotic events compared to patients without these conditions.^1^ In orthopaedic surgery, studies have shown that patients with APS undergoing total hip or knee arthroplasty experience elevated rates of deep vein thrombosis (DVT), pulmonary embolism (PE), and stroke.^9,17^

Although thromboembolic complications are less frequently reported following foot and ankle procedures than after hip or knee arthroplasty, such events do occur and may be underrecognized in the APS population.^2,8^ To date, no studies have directly compared postoperative complication rates between patients with and without APS undergoing foot and ankle surgery, leaving the impact of APS in this surgical setting unclear.

The primary objective of this study is to compare postoperative complication rates, including thromboembolic events, between patients with and without APS undergoing foot and ankle surgery. We hypothesize that APS is associated with higher rates of thromboembolic complications. Our findings aim to clarify the risks associated with APS in foot and ankle surgery and contribute to informed perioperative management and thromboprophylaxis strategies.

## Methods

### Data source

This retrospective study used data collected on June 21, 2025, from the TriNetX Research Network, which provided access to electronic medical records (diagnoses, procedures, medications, laboratory values, genomic information) from approximately 140 million patients from 104 health care organizations. Data are acquired as discrete data elements or from natural language processing of medical notes and reports syntactically harmonized into TriNetX servers followed by semantic harmonization to ensure consistency of data definitions. This study is exempt from Institutional Review Board approval. The data reviewed is a secondary analysis of existing data, does not involve intervention or interaction with human subjects, and is deidentified per the deidentification standard defined in Section §164.514(a) of the HIPAA Privacy Rule.

### Cohort Selection

The TriNetX Research Database was queried using the *International Classification of Diseases, Tenth Revision* (*ICD-10*) and *Current Procedural Terminology* (*CPT*) codes to identify patients the underwent foot and ankle surgery (Codes in Supplemental 1) between March 25, 2004, and June 19, 2025. This population was divided into 2 cohorts: patients that had a diagnosis of APS (*ICD-10*: D68.61) within 5 years prior to the procedure (APS) and patients that did not have an APS diagnosis (control). These patients were further stratified based on whether they received VTE prophylaxis within 1 month postoperatively, resulting in 4 cohorts: APS+VTE, APS−VTE, control+VTE, and control−VTE. VTE prophylaxis was defined as any of the following: aspirin (81 or 325 mg), warfarin, fondaparinux (5 or 12.5 mg/mL), dabigatran etexilate (75 or 150 mg), enoxaparin (100 mg/mL), rivaroxaban (10 mg or 15 mg), heparin (1000 or 5000 U/mL). The query was configured to include patients prescribed these medications any time between 1 day and 1 month postoperatively. Patients with less than 3 months of follow-up were excluded.

### Propensity Matching

Propensity score matching was performed to reduce the impact of confounding variables and ensure balanced comparisons between cohorts. Patients were matched in a 1:1 ratio using greedy nearest-neighbor matching with a caliper of 0.1 pooled SDs, and propensity scores were estimated using logistic regression. The covariates included in the matching process were age, sex, body mass index (BMI), tobacco use, procedure type and select comorbidities. Comorbidities were selected to reflect the Charlson Comorbidity Index (CCI), which was calculated as a measure of total comorbidity burden for each cohort. CCI scores were calculated according to CCI standards; however, because of limitations of TriNetX database, the weight assigned to each condition was not adjusted for disease severity (Supplemental Table 2). Matching was conducted with a tolerance of 0.01 to minimize discrepancies in propensity scores between matched pairs. Covariate balance was evaluated with standardized mean differences (SMDs) after matching. SMD of less than 0.1 was considered sufficient for covariate balance, as recommended. As per TriNetX privacy policy, counts of less than 10 patients are recorded as “<10” rather than providing an exact number, in order to protect patient anonymity and prevent reidentification.

### Data Collection and Statistical Analysis

Patient outcomes were identified using *ICD-10* and *CPT* codes. The incidence of pulmonary embolism, deep vein thrombosis, anemia, and stroke were analyzed at 30 and 90 days postoperatively. Additionally, preoperative laboratory test values were compared between cohorts. Statistical analysis was performed using the TriNetX platform, which uses Java, R, and Python for statistical computing. For all outcomes, risk differences with *P* values were reported. TriNetX calculates *P* values using Student *t* tests for continuous variables and *z* tests for categorical variables. Additionally, risk ratios (RRs) were calculated for all outcomes to demonstrate relative risk. Benjamini-Hochberg false discovery rate was calculated for each analysis to set the significance level.

## Results

A total of 576 571 patients with foot and ankle surgeries were identified. From this cohort, a total of 1468 patients in the APS cohort and 575 103 patients in the control cohort were identified. Among the surgeries in the APS cohort, 22.6% were fracture/dislocation procedures, 50.7% were revision/reconstruction procedures, 13.1% were excision procedures, and 13.6% were arthrodesis procedures (Table 1). Among the surgeries in the Control cohort, 33.8% were fracture/dislocation procedures, 45.1% were revision/reconstruction procedures, 11.0% were excision procedures, and 10.1% were arthrodesis procedures (Table 1).

**Table 1.** Procedure Mix Percentages for APS and Control Cohorts.

Procedure	APS, %	Control, %
Trauma		
Fracture and/or dislocation procedures on the leg (tibia and fibula) and ankle joint	17.5	26.9
Fracture and/or dislocation procedures on the foot and toes	5.1	6.9
Elective		
Repair, revision, and/or reconstruction procedures on the foot and toes	31.4	25.2
Repair, revision, and/or reconstruction procedures on the leg (tibia and fibula) and ankle joint	19.3	19.9
Excision procedures on the foot and toes	13.1	11.0
Arthrodesis procedures on the foot and toes	12.0	8.7
Arthrodesis procedures on the leg (tibia and fibula) and ankle joint	1.6	1.4

Abbreviation: APS, antiphospholipid syndrome.

### APS+VTE vs Control+VTE

There were 531 patients in the APS+VTE cohort and 131 219 patients in the control+VTE cohort. Prior to matching, patients in the APS+VTE cohort were older (56.1 vs 50.8 years, *P* < .001) and had a significantly higher burden of comorbidities compared to the control+VTE cohort, as reflected by a higher mean CCI (2.1 vs 0.9). They were more likely to be female (62.7% vs 46.5%, *P* < .001) and had significantly higher rates of diabetes mellitus (25.0% vs 18.3%, *P* < .001), renal disease (19.0% vs 8.0%, *P* < .001), pulmonary disease (28.6% vs 15.5%, *P* < .001), and congestive heart failure (13.7% vs 5.8%, *P* < .001), among other conditions. They also had significantly higher rates of obesity, with 17.5% having a BMI >40 compared with 9.7% in the control cohort (*P* < .001). After matching, baseline characteristics were well balanced between APS+VTE and control+VTE cohorts (n = 524 per group), including comorbidities, BMI categories, and sex distribution (Table 2).

**Table 2.** Demographics for APS+VTE and Control+VTE Cohorts Before and After Propensity Matching.

Characteristics	Before Matching			After Matching			Standard Mean Difference
	APS +VTE (n = 531)	Control +VTE (n = 131 219)	*P* Value	APS +VTE (n = 524)	Control +VTE (n = 524)	*P* Value	
Mean age, y	56.1	50.8	<.001*	56	57.2	.234	0.074
Male, n (%)	140 (26.4)	64 304 (49.0)	<.001*	139 (26.5)	128 (24.4)	.435	0.016
Female, n (%)	333 (62.7)	61 045 (46.5)	<.001*	330 (63.0)	334 (63.7)	.798	0.048
Acute myocardial infarction, n (%)	14 (2.6)	2164 (1.6)	.075	14 (2.7)	<10 (1.9)^a^	.409	0.017
Cancer, n (%)	128 (24.1)	12 638 (9.6)	<.001*	123 (23.5)	142 (27.1)	.177	0.009
Cerebral vascular accident, n (%)	41 (7.7)	2135 (1.6)	<.001*	41 (7.8)	33 (6.3)	.335	0.083
Congestive heart failure, n (%)	73 (13.7)	7599 (5.8)	<.001*	71 (13.6)	71 (13.6)	>.999	0.039
Connective tissue disorder, n (%)	78 (14.7)	1774 (1.4)	<.001*	73 (13.9)	75 (14.3)	.859	0.010
Dementia, n (%)	<10 (1.9)^a^	1374 (1.0)	.059	<10 (1.9)^a^	<10 (1.9)^a^	>.999	0.035
Diabetes mellitus, n (%)	133 (25.0)	24 078 (18.3)	<.001*	130 (24.8)	132 (25.2)	.887	0.011
Hemiplegia, n (%)	13 (2.4)	505 (0.4)	<.001*	13 (2.5)	<10 (1.9)^a^	.527	0.087
HIV, n (%)	0 (0.0)	492 (0.4)	.157	0 (0.0)	0 (0.0)	–	0.000
Liver disease, n (%)	47 (8.9)	5500 (4.2)	<.001*	45 (8.6)	46 (8.8)	.913	0.068
Peptic ulcer, n (%)	<10 (1.9)^a^	480 (0.4)	<.001*	<10 (1.9)^a^	<10 (1.9)^a^	>.999	0.007
Peripheral vascular disease, n (%)	67 (12.6)	5468 (4.2)	<.001*	63 (12.0)	75 (14.3)	.273	0.060
Pulmonary disease, n (%)	152 (28.6)	20 287 (15.5)	<.001*	148 (28.2)	144 (27.5)	.783	0.051
Renal disease, n (%)	101 (19.0)	10 471 (8.0)	<.001*	97 (18.5)	99 (18.9)	.874	0.039
Tobacco use, n (%)	105 (19.8)	26 135 (19.9)	.934	102 (19.5)	94 (17.9)	.526	0.000
Deep venous thromboembolism, n (%)	102 (19.2)	2802 (2.1)	<.001*	96 (18.3)	89 (17.0)	.571	0.000
Pulmonary embolism, n (%)	78 (14.7)	1943 (1.5)	<.001*	72 (13.7)	57 (10.9)	.158	0.087
Mean CCI	2.1	0.9		2	2.1		
BMI, n (%)							
0-19	18 (3.4)	5194 (4.0)	.502	18 (3.4)	20 (3.8)	.741	0.020
20-29	216 (40.7)	52 930 (40.3)	.873	214 (40.8)	219 (41.8)	.754	0.019
30-39	246 (46.3)	39 046 (29.8)	<.001*	243 (46.4)	241 (46.0)	.901	0.008
>40	93 (17.5)	12 668 (9.7)	<.001*	93 (17.7)	108 (20.6)	.239	0.073
Trauma, n (%)							
Fracture and/or dislocation procedures on the leg (tibia and fibula) and ankle joint	168 (31.6)	56 891 (43.4)	<.001*	168 (32.1)	168 (32.1)	>.999	0.000
Fracture and/or dislocation procedures on the foot and toes	34 (6.4)	11 592 (8.8)	.049	32 (6.1)	35 (6.7)	.705	0.023
Elective, n (%)							
Repair, revision and/or reconstruction procedures on the foot and toes	120 (22.6)	9394 (7.2)	<.001*	117 (22.3)	130 (24.8)	.344	0.058
Repair, revision, and/or reconstruction procedures on the leg (tibia and fibula and ankle joint	116 (21.8)	18 514 (14.1)	<.001*	110 (21.0)	115 (21.9)	.707	0.023
Excision procedures on the foot and toes	62 (11.7)	6473 (4.9)	<.001*	61 (11.6)	59 (11.3)	.846	0.012
Arthrodesis procedures on the foot and toes	57 (10.7)	8640 (6.6)	<.001*	57 (10.9)	66 (12.6)	.388	0.053
Arthrodesis procedures on the leg (tibia and fibula) and ankle joint	13 (2.4)	2907 (2.2)	.716	11 (2.1)	11 (2.1)	>.999	0.000

Abbreviations: APS, antiphospholipid syndrome; APS+VTE, APS patients receiving VTE prophylaxis; BMI, body mass index; CCI, Charlson Comorbidity Index; Control+VTE, patients without APS receiving VTE prophylaxis; HIV, Human immunodeficiency virus, VTE, venous thromboembolism.

a TriNetX does not provide exact numbers if less than 10 to protect against identification.

* *P* < .003.

The APS+VTE cohort exhibited similar rates of strokes compared to matched controls at both 30 days (4.6% vs 2.9%, *P* = .142, RR = 1.60) and 90 days (6.3% vs 3.8%, *P* = .067, RR = 1.65) postoperatively. However, patients in the APS+VTE cohort experienced significantly higher rates of deep vein thrombosis at 90 days (13.4% vs 9.0%, *P* = .024, RR = 1.49), but not 30 days (8.8% vs 6.9%, *P* = .250, RR = 1.28). Rates of pulmonary embolism were the same at 30 days (4.8% vs 4.8%, *P* > .99, RR = 1.00) and 90 days (6.3% vs 6.3%, *P* > .99, RR = 1.00). Interestingly, anemia was significantly less common in the APS+VTE group at 30 days (14.7% vs 22.3%, *P* = .001, RR = 0.66) and 90 days (16.4% vs 23.9%, *P* = .003, RR = 0.69) (Table 3).

**Table 3.** Thirty- and 90-Day Complications for APS+VTE (n = 524) and Control+VTE (n = 524) Cohorts.

	APS+VTE, n (%)	Control+VTE, n (%)	Risk Difference (%)	*P* Value	Risk Ratio	95% CI
Pulmonary embolism						
30 d	25 (4.8)	25 (4.8)	0	>.999	1	0.58-1.72
90 d	33 (6.3)	33 (6.3)	0	>.999	1	0.63-1.6
Anemia						
30 d	77 (14.7)	117 (22.3)	–7.6	.001*	0.66	0.51-0.85
90 d	86 (16.4)	125 (23.9)	–7.4	.003*	0.69	0.54-0.88
Stroke						
30 d	24 (4.6)	15 (2.9)	1.7	.142	1.6	0.85-3.02
90 d	33 (6.3)	20 (3.8)	2.5	.067	1.65	0.96-2.84
Deep vein thrombosis						
30 d	46 (8.8)	36 (6.9)	1.9	.250	1.28	0.84-1.94
90 d	70 (13.4)	47 (9.0)	4.4	.024*	1.49	1.05-2.11

Abbreviations: APS, antiphospholipid syndrome; APS+VTE, APS patients receiving VTE prophylaxis; Control+VTE, patients without APS receiving VTE prophylaxis; VTE, venous thromboembolism.

* Benjamini-Hochberg false discovery rate was calculated to identify significance level of *P* ≤ .013 for 30 days and *P* ≤ .025 for 90 days.

### APS–VTE vs Control–VTE

There were 937 patients in the APS–VTE cohort and 443 884 patients in the control–VTE cohort. Prior to matching, patients in the APS–VTE cohort were older (52.7 vs 46.3 years, *P* < .001) and had a significantly higher comorbidity burden, as reflected by a higher mean CCI (1.8 vs 0.7). They were more likely to be female (69.3% vs 55.9%, *P* < .001) and had significantly higher rates of diabetes mellitus (19.5% vs 11.5%, *P* < .001), renal disease (11.3% vs 3.7%, *P* < .001), pulmonary disease (24.7% vs 12.6%, *P* < .001), and congestive heart failure (6.5% vs 2.2%, *P* < .001). Additionally, obesity was more common, with 12.2% having a BMI >40 compared to 7.1% in controls (*P* < .001). After matching, baseline characteristics were well balanced between the APS–VTE and control–VTE cohorts (n = 932 per group), including age, sex, BMI, and comorbidities (Supplemental Table 3).

The APS–VTE cohort exhibited similar incidence of stroke compared with matched controls at both 30 days (1.5% vs 1.1%, *P* = .411, RR = 1.40) and 90 days (2.4% vs 1.8%, *P* = .418, RR = 1.29) postoperatively. Patients in the APS–VTE cohort experienced similar rates of pulmonary embolism at 30 days (3.8% vs 2.3%, *P* = .057, RR = 1.67), but significantly higher rates at 90 days (5.8% vs 3.3%, *P* = .011, RR = 1.74). Rates of deep vein thrombosis were similar at 30 days (3.9% vs 4.0%, *P* = .905, RR = 0.97), and 90 days (6.8% vs 6.0%, *P* = .507, RR = 1.12). Rates of anemia were comparable at 30 days (4.3% vs 3.5%, *P* = .403, RR = 1.21), and 90 days (5.0% vs 4.1%, *P* = .318, RR = 1.24) (Table 4).

**Table 4.** Thirty- and 90-Day Complications for APS–VTE (n = 932) and Control–VTE (n = 932) Cohorts.

	APS–VTE n (%)	Control–VTE n (%)	Risk Difference (%)	*P* Value	Risk Ratio	95% CI
Pulmonary embolism						
30 d	35 (3.8)	21 (2.3)	1.5	.057	1.67	0.98-2.84
90 d	54 (5.8)	31 (3.3)	2.5	.011*	1.74	1.13-2.68
Anemia						
30 d	40 (4.3)	33 (3.5)	0.8	.403	1.21	0.77-1.9
90 d	47 (5.0)	38 (4.1)	1	.318	1.24	0.81-1.88
Stroke						
30 d	14 (1.5)	<10 ^a^ (1.1)	0.4	.411	1.4	0.63-3.14
90 d	22 (2.4)	17 (1.8)	0.5	.418	1.29	0.69-2.42
Deep vein thrombosis						
30 d	36 (3.9)	37 (4.0)	-0.1	.905	0.97	0.62-1.53
90 d	63 (6.8)	56 (6.0)	0.8	.507	1.12	0.79-1.59

Abbreviations: APS, antiphospholipid syndrome; APS–VTE, APS patients without postoperative VTE prophylaxis; Control–VTE, patients without APS and VTE prophylaxis; VTE, venous thromboembolism.

a TriNetX does not provide exact numbers if less than 10 to protect against identification.

* Benjamini-Hochberg false discovery rate was calculated to identify significance level of *P* ≤ .013 for 30 and 90 days.

Among all APS patients, 340 individuals had not received any anticoagulation therapy in the 6 months prior to surgery. This subgroup was compared to matched controls to evaluate differences in laboratory values obtained during the same preoperative period. The APS cohort demonstrated significantly higher mean International Normalized Ratio (INR) (1.64 vs 1.09, *P* < .001), activated partial thromboplastin time (aPTT) (38.5 vs 29.2, *P* < .001), and prothrombin time (PT) (17.7 vs 12.3, *P* < .001). Additionally, estimated dry weight (EDW) (14.3 vs 13.7, *P* < .001) and serum creatinine were significantly higher (1.08 vs 0.94, *P* < .001), along with urea nitrogen (17.0 vs 15.9, *P* < .001), potassium (4.14 vs 4.10, *P* = .012) and bicarbonate (26.2 vs 25.8, *P* < .001) (Supplemental Table 4).

## Discussion

In this matched retrospective cohort study, we found significantly higher complication rates in patients with antiphospholipid syndrome (APS) undergoing foot and ankle surgery with and without postoperative VTE prophylaxis. Compared with the control+VTE cohort, patients in the APS+VTE cohort had significantly higher rates of deep vein thrombosis at 90 days postoperatively, but had significantly lower rates of anemia at 30 and 90 days. Patients in the APS–VTE cohort had higher rates of pulmonary embolism at 90 days. Additionally, a preoperative laboratory analysis of APS patients not on anticoagulation revealed significantly higher INR, aPTT, and PT values, along with lower platelet counts and higher serum creatinine, compared with controls. To our knowledge, this study represents the first time that foot and ankle surgery patients with APS have been extensively studied in the literature to date.

### Thromboembolic Disease

Foot and ankle surgery patients with APS had an increased rate of thromboembolic disease, including PE and DVT, compared with matched controls, irrespective of postoperative anticoagulation use. This elevated risk aligns with the known prothrombotic state associated with APS and is consistent with findings reported in prior studies. A recent retrospective study of patients undergoing total hip arthroplasty and total knee arthroplasty demonstrated increased rates of deep vein thrombosis and pulmonary embolism in patients with APS compared with matched controls.^9^ Another retrospective study by Zhang et al^17^ using the PearlDiver database demonstrated significantly higher rates of stroke and deep vein thrombosis in APS patients undergoing total knee arthroplasty.

Our study extends previous research by evaluating venous thromboembolism (VTE) rates in patients with APS both with and without the use of postoperative anticoagulation. While some studies in the surgical literature have reiterated the importance of tight control of anticoagulation in the perioperative period as that can decrease both thrombotic and hemorrhagic complications, our findings suggest that postoperative anticoagulation alone may not fully mitigate the elevated VTE risk associated with APS.^3,6^ However, an overall scarcity of orthopaedic literature exists examining the impact of APS on orthopaedic surgery patients.

### Anemia

In this study, APS patients who received postoperative anticoagulation had significantly lower rates of anemia compared to matched controls, whereas APS patients who did not receive anticoagulation exhibited similar anemia rates to their matched counterparts. In contrast, Hirpara et al^9^ reported higher rates of anemia in APS patients undergoing total knee or hip arthroplasty compared to controls; however, that study did not differentiate between patients who did and did not receive postoperative anticoagulation. The lower anemia rates observed in our anticoagulated APS cohort may reflect differential effects of anticoagulation in patients with and without APS. Specifically, patients without APS and who are not inherently hypercoagulable may be more susceptible to bleeding complications when exposed to aggressive anticoagulation regimens. However, our analysis did not evaluate specific anticoagulant dosing, and it remains unclear whether dosing strategies differed between APS and control groups.

### Preoperative Risk Stratification and Optimization

Lastly, in this study, we examined a standard set of orthopaedic preoperative laboratory tests, including complete blood count (CBC), coagulation studies, and basic metabolic panel (BMP) to identify differences in patients with APS. In this study, we found that patients with APS had significantly increased PT, aPTT, and INR. Similarly, previous studies of APS patients have demonstrated prolonged PT and INR compared with the general population. Notably, despite being in a hypercoagulable state, APS patients may have falsely elevated PT and INR, because of the presence of antiphospholipid antibodies, most commonly lupus anticoagulant, which can alter test results.^5,11^ Furthermore, patients in the APS cohort had a significantly elevated creatinine level, however, the mean value was still within the normal range. Patients in the APS cohort also showed elevated estimated dry weight (EDW), sodium, urea nitrogen, potassium, and bicarbonate levels. Preoperative laboratory abnormalities, specifically coagulation studies and platelet counts, may prompt further evaluation by specialists before orthopaedic surgery when possible.

### Recommendations

Given the elevated risks of medical and surgical complications in patients with antiphospholipid syndrome, our findings support existing knowledge of APS as a high-risk population in the perioperative setting. Although this study reinforces the importance of heightened clinical vigilance, it does not provide specific guidance on preoperative screening protocols or procedure-specific management strategies. As such, the findings should be interpreted as exploratory and hypothesis-generating, rather than practice-changing. Nevertheless, orthopaedic surgeons may want to consider employing a multidisciplinary team-based approach to care for APS patients. Especially in the setting of elective procedures, this may include additional preoperative clearances by cardiology, rheumatology, and hematology, in addition to the patient’s primary care physician.

A review article by Kim et al^10^ discussing the anesthetic and medical considerations for patients with APS undergoing noncardiac surgery highlights the importance of preoperative lifestyle changes aimed at reducing cardiovascular risk factors in patients with APS. This includes smoking cessation, weight loss, and control of hypertension and hyperlipidemia. Additionally, this study recommended that although only high-risk patients with APS require thromboprophylaxis at all times, patients in high-risk situations such as surgery, prolonged immobilization, and puerperium should also receive some form of VTE chemoprophylaxis.^10^ More recently, some authors have also advocated for the incorporation of adjunct therapies, such as antiplatelet and antirheumatic medications, to address the complement-mediated inflammatory pathways associated with APS.^14,16^ Despite its low prevalence, APS may go undiagnosed prior to surgery, as standard preoperative screening protocols do not universally include coagulation studies such as PT or INR; therefore, heightened clinical awareness and thorough history-taking remain critical. However, definitive recommendations regarding screening or perioperative management cannot be made based on this study alone.

### Clinical Relevance

Although APS represents only 1 specific hypercoagulable condition, this study's findings provide broader insights applicable to perioperative management of other hypercoagulable states, such as malignancy-associated thrombophilia and inherited thrombophilias. By demonstrating that postoperative anticoagulation alone may not fully mitigate thrombotic risk in APS, this study raises important considerations regarding anticoagulation efficacy in other hypercoagulable patient populations.

### Limitations

This study has various limitations. The retrospective nature of the study could introduce selection bias. Additionally, the TriNetX database aggregates information from multiple centers, so there could be significant heterogeneity in data collection protocols. Identification of patients with APS relied on *ICD-10* coding rather than laboratory confirmation, which raises the possibility of diagnostic misclassification. APS is a clinical diagnosis requiring persistently positive antiphospholipid antibody testing. However, not all contributing health care organizations in TriNetX share laboratory data, and restricting the cohort to patients with available antibody test results would have excluded a large proportion of APS cases and limited the generalizability of the findings. Although our *ICD*-based strategy has been employed in prior studies to identify APS patients, we acknowledge the lack of antibody profile, laboratory confirmation, and autoimmune disease details as a key limitation of our case definition.^9,17^

Additionally, this study did not elicit differences in surgical approach, perioperative medical management of antiphospholipid syndrome, and the presence or absence of other rheumatologic abnormalities. The postoperative VTE prophylaxis protocol could not be abstracted from the TriNetX database, meaning neither length of prophylaxis nor frequency of administration can be commented on. Moreover, in this study, we were unable to examine the underlying biologic effects of antiphospholipid syndrome and thus cannot definitively identify specific processes that make APS patients more predisposed to VTE and wound complications. Additionally, this study includes a heterogeneous mix of foot and ankle procedures with varying baseline risks of thromboembolic and surgical complications. Although stratification by procedure type could offer greater specificity, subgroup analyses were underpowered because of limited sample sizes, particularly in the APS cohort. As such, the pooled analysis may limit the applicability of findings to specific surgical subtypes. Future studies with larger sample sizes should compare VTE risk across specific surgical procedures and evaluate the effects of surgical approach and comorbid rheumatologic conditions in APS patients.

## Conclusion

In this study, foot and ankle surgery patients with APS demonstrated significantly increased thromboembolic events compared with matched controls, regardless of postoperative anticoagulation. This aligns with prior orthopaedic studies and indicates that standard anticoagulation alone may not fully address the thrombotic risks associated with APS. Additionally, APS patients who received postoperative anticoagulation had significantly lower anemia rates than matched controls, potentially reflecting differential bleeding risks between hypercoagulable APS patients and controls. Given the elevated perioperative risks in APS patients, a multidisciplinary approach emphasizing careful preoperative assessment, lifestyle optimization, and vigilant thromboprophylaxis may be beneficial. Although focused on APS, this study highlights that postoperative anticoagulation may be insufficient in hypercoagulable states more broadly, offering insights relevant to the perioperative management of other conditions such as malignancy-associated and inherited thrombophilias.

## Supplemental Material

sj-pdf-1-fao-10.1177_24730114251393650 – Supplemental material for Antiphospholipid Syndrome in Orthopaedic Foot and Ankle Surgery: A Propensity-Matched Analysis Supplemental material, sj-pdf-1-fao-10.1177_24730114251393650 for Antiphospholipid Syndrome in Orthopaedic Foot and Ankle Surgery: A Propensity-Matched Analysis by Kush Mody, Avani A. Chopra, Michael Greenberg, Darian Napoleon, Tyler Stewart, Michael Aynardi and Sheldon Lin in Foot & Ankle Orthopaedics

## Appendix

**Supplemental Table 1.** Query Codes.

Diagnosis or Procedure	Code
Foot and ankle surgery	*CPT*: 27814, 27829, 1005272, 27792, 27759, 1005278, 27758, 27766, 27784, 1005290, 27769, 27832, 1005194, 1005295, 1005386, 28485, 1014598, 28615, 28445, 28645, 28446, 28465, 28555, 28585, 1005511, 1005348, 1005359, 1014596, 28080, 28110, 28160, 1005345, 1005341, 28126, 28153, 28116, 28140, 28150, 28035 *ICD-10-PCS*: 0SRP, 0SRQ, 0SRF, 0SRG, 0SRH, 0SRJ, 0SRK, 0SRL, 0SRM, 0SRN, 0LQN0ZZ, 0LQP0ZZ, 0LQS0ZZ, 0LQT0ZZ, 0LQV0ZZ, 0LQW0ZZ, 0LXN0ZZ, 0LXP0ZZ, 0LXS0ZZ, 0LXT0ZZ, 0LXV0ZZ, 0LXW0ZZ, 0QSG04Z, 0QSG06Z, 0QSG0ZZ, 0QSH04Z, 0QSH06Z, 0QSH0ZZ, 0QSJ04Z, 0QSJ06Z, 0QSJ0ZZ, 0QSK04Z, 0QSK06Z, 0QSK0ZZ, 0QSL04Z, 0QSL0ZZ, 0QSM04Z, 0QSM0ZZ, 0QSN042, 0QSN04Z, 0QSN0Z2, 0QSN0ZZ, 0QSP042, 0QSP04Z, 0QSP0Z2, 0QSP0ZZ, 0SGF03Z, 0SGF04Z, 0SGF07Z, 0SGF0JZ, 0SGF0KZ, 0SGF0ZZ, 0SGG03Z, 0SGG04Z, 0SGG07Z, 0SGG0JZ, 0SGG0KZ, 0SGG0ZZ, 0SGH03Z, 0SGH04Z, 0SGH07Z, 0SGH0JZ, 0SGH0KZ, 0SGH0ZZ, 0SGJ03Z, 0SGJ04Z, 0SGJ05Z, 0SGJ07Z, 0SGJ0JZ, 0SGJ0KZ, 0SGJ0ZZ, 0SGK03Z, 0SGK04Z, 0SGK07Z, 0SGK0JZ, 0SGK0KZ, 0SGK0ZZ, 0SGL03Z, 0SGL04Z, 0SGL07Z, 0SGL0JZ, 0SGL0KZ, 0SGL0ZZ, 0SGM03Z, 0SGM04Z, 0SGM07Z, 0SGM0JZ, 0SGM0KZ, 0SGM0ZZ, 0SGN03Z, 0SGN04Z, 0SGN07Z, 0SGN0JZ, 0SGN0KZ, 0SGN0ZZ, 0SGP03Z, 0SGP04Z, 0SGP07Z, 0SGP0JZ, 0SGP0KZ, 0SGP0ZZ, 0SGQ03Z, 0SGQ04Z, 0SGQ07Z, 0SGQ0JZ, 0SGQ0KZ, 0SGQ0ZZ, 0SSF04Z, 0SSF0ZZ, 0SSG04Z, 0SSG0ZZ, 0SSH04Z, 0SSH0ZZ, 0SSJ04Z, 0SSJ0ZZ, 0SSK04Z, 0SSK0ZZ, 0SSL04Z, 0SSL0ZZ
Antiphospholipid syndrome	*ICD-10-CM*: D68.61
ED visits	*CPT*: 99281, 99282, 99283, 99284, 99285, 99288
Wound complications	*ICD-10-CM*: T81.30, T81.31, T81.32, T81.41, T81.42, T81.43, T81.49
Pulmonary embolism	*ICD-10-CM*: I26
Deep vein thrombosis	*ICD-10-CM*: I82.4
Stroke	*ICD-10-CM*: I63
Minor adverse events	*ICD-10-CM*: J12, J13, J14, J15, J16, J17, J18, N17, N39.0, D62 *ICD-10-PCS*: 302
Severe adverse events	*ICD-10-CM*: A40, A41, I21, I46

Abbreviations: ED, emergency department; *ICD-10-CM, International Classification of Disease, Tenth Revision–Clinical Modification; ICD-10-PCS, International Classification of Disease, Tenth Revision–Procedure Coding System.*

**Supplemental Table 2.** *ICD-10* Coding for Modified CCI.^a^

Condition	*ICD-10* Code	Weight
Acute myocardial infarction	I21	1
Congestive heart failure	I50	1
Peripheral vascular disease	I73	1
Cerebral vascular accident	I63	1
Dementia	F03	1
Pulmonary disease	J40-J4A	1
Connective tissue disorder	M30-M36	1
Peptic ulcer	K25	1
Liver disease	K70-K77	1
Diabetes mellitus	E08-E13	1
Hemiplegia	G81.9	2
Renal disease	N18	2
Cancer	C00-D49	2
HIV	B20	6

Abbreviations: CCI, Charlson Comorbidity Index; HIV, Human immunodeficiency virus, *ICD-10, International Classification of Disease, Tenth Revision.*

a CCI equals the sum of the weighted score. Because of limitations of the TriNetX database, severity of the condition was not included.

**Supplemental Table 3.** Demographics for APS–VTE and Control–VTE Cohorts Before and After Propensity Matching

Characteristics	Before Matching			After Matching			Standard Mean Difference
	APS–VTE (n = 937)	Control–VTE (n = 443 884)	*P* Value	APS–VTE (n = 932)	Control–VTE (n = 932)	*P* Value	
Mean age, y	52.7	46.3	<.001*	52.7	53.1	.605	0.024
Male, n (%)	212 (22.6)	185 260 (41.7)	<.001*	210 (22.5)	199 (21.3)	.538	0.009
Female, n (%)	649 (69.3)	248 156 (55.9)	<.001*	648 (69.5)	644 (69.0)	.841	0.029
Acute myocardial infarction, n (%)	15 (1.6)	2435 (0.5)	<.001*	15 (1.6)	11 (1.2)	.430	0.012
Cancer, n (%)	226 (24.1)	47 621 (10.7)	<.001*	225 (24.1)	246 (26.4)	.263	0.052
Cerebral vascular accident, n (%)	56 (6.0)	3250 (0.7)	<.001*	56 (6.0)	49 (5.3)	.482	0.044
Congestive heart failure, n (%)	61 (6.5)	9580 (2.2)	<.001*	60 (6.4)	53 (5.7)	.497	0.034
Connective tissue disorder, n (%)	164 (17.5)	6327 (1.4)	<.001*	160 (17.1)	172 (18.4)	.468	0.016
Dementia, n (%)	<10 (1.1)^a^	1294 (0.3)	<.001*	<10 (1.1) ^a^	<10 (1.1) ^a^	>.999	0.006
Diabetes mellitus, n (%)	183 (19.5)	51 237 (11.5)	<.001*	182 (19.5)	166 (17.8)	.342	0.068
Hemiplegia, n (%)	14 (1.5)	1631 (0.4)	<.001*	14 (1.5)	14 (1.5)	>.999	0.033
HIV, n (%)	<10 (1.1) ^a^	1315 (0.3)	<.001*	<10 (1.1) ^a^	<10 (1.1) ^a^	>.999	0.097
Liver disease, n (%)	72 (7.7)	11 407 (2.6)	<.001*	70 (7.5)	72 (7.7)	.861	0.008
Peptic ulcer, n (%)	13 (1.4)	1287 (0.3)	<.001*	13 (1.4)	<10 (1.1) ^a^	.529	0.031
Peripheral vascular disease, n (%)	94 (10.0)	10 277 (2.3)	<.001*	92 (9.9)	83 (8.9)	.475	0.033
Pulmonary disease, n (%)	231 (24.7)	55 979 (12.6)	<.001*	229 (24.5)	234 (25.1)	.789	0.037
Renal disease, n (%)	106 (11.3)	16 599 (3.7)	<.001*	103 (11.0)	84 (9.0)	.143	0.000*
Tobacco use, n (%)	125 (13.3)	49 769 (11.2)	.039	124 (13.3)	126 (13.5)	.892	0.029
Acute embolism and thrombosis of deep veins of lower extremity, n (%)	135 (14.4)	3462 (0.8)	<.001*	131 (14.0)	126 (13.5)	.737	0.000*
Pulmonary embolism, n (%)	94 (10.0)	2127 (0.5)	<.001*	91 (9.8)	66 (7.1)	.037	0.000*
Mean CCI	1.8	0.7		1.7	1.7		
BMI							
0-19	61 (6.5)	25 433 (5.7)	.305	61 (6.5)	72 (7.7)	.322	0.046
20-29	424 (45.3)	180 532 (40.7)	.004	423 (45.3)	428 (45.9)	.816	0.011
30-39	387 (41.3)	117 793 (26.5)	<.001*	384 (41.2)	392 (42.0)	.707	0.017
>40	114 (12.2)	31 647 (7.1)	<.001*	113 (12.1)	117 (12.5)	.778	0.013
Trauma							
Fracture and/or dislocation procedures on the leg (tibia and fibula) and ankle joint, n (%)	119 (12.7)	98 753 (22.2)	<.001*	119 (12.8)	104 (11.1)	.284	0.050
Fracture and/or dislocation procedures on the foot and toes, n (%)	49 (5.2)	28 500 (6.4)	.137	49 (5.3)	63 (6.8)	.172	0.063
Elective							
Repair, revision, and/or reconstruction procedures on the foot and toes, n (%)	395 (42.2)	136 701 (30.8)	<.001*	392 (42.0)	386 (41.4)	.778	0.013
Repair, revision, and/or reconstruction procedures on the leg (tibia and fibula) and ankle joint, n (%)	201 (21.5)	96 784 (21.8)	.794	200 (21.4)	214 (22.9)	.435	0.036
Excision procedures on the foot and toes	152 (16.2)	57 240 (12.9)	.002	152 (16.3)	153 (16.4)	.950	0.003
Arthrodesis procedures on the foot and toes, n (%)	140 (14.9)	41 532 (9.4)	<.001*	139 (14.9)	158 (16.9)	.229	0.056
Arthrodesis procedures on the leg (tibia and fibula) and ankle joint, n (%)	14 (1.5)	5188 (1.2)	.355	14 (1.5)	12 (1.3)	.693	0.018

Abbreviations: APS, antiphospholipid syndrome; APS–VTE, APS patients without postoperative VTE prophylaxis; BMI, body mass index; CCI, Charlson Comorbidity Index; Control–VTE, patients without APS and VTE prophylaxis; HIV, Human immunodeficiency virus; VTE, venous thromboembolism.

a TriNetX does not provide exact numbers if less than 10 to protect against identification.

* *P* < .003.

**Supplemental Table 4.** Comparison of Preoperative Laboratory test Values between APS and Control Cohorts.

Laboratory test	APS	Control	*P* Value
INR	1.64	1.09	<.001*
aPTT	38.51	29.19	<.001*
PT	17.69	12.28	<.001*
Hematocrit	38.58	38.81	.237
Hemoglobin	12.76	12.95	.004*
Platelets	252.29	258.16	.029*
EDW	14.29	13.74	<.001*
Phosphate	3.48	3.42	.334
Magnesium	1.95	1.94	.363
Calcium	9.16	9.18	.413
Glucose	109.95	112.31	.065
Creatinine	1.08	0.94	<.001*
Urea nitrogen	16.96	15.93	<.001*
Bicarbonate	26.19	25.78	<.001*
Chloride	103.10	103.26	.162
Potassium	4.14	4.10	.012*
Sodium	139.13	138.83	.001*

Abbreviations: APS, antiphospholipid syndrome; aPTT, activated partial thromboplastin time; EDW, estimated dry weight; INR, international normalized ratio; PT, prothrombin time; PTT, partial thromboplastin time.

* Benjamini-Hochberg false discovery rate was calculated to identify significance level of *P* ≤ .029.
